# Response: Commentary: Sensitivity, Specificity, and Predictive Values: Foundations, Pliabilities, and Pitfalls in Research and Practice

**DOI:** 10.3389/fpubh.2019.00408

**Published:** 2020-01-14

**Authors:** Robert Trevethan

**Affiliations:** Independent Researcher, Albury, NSW, Australia

**Keywords:** screening, diagnosis, sensitivity, specificity, predictive values, predictive summary index, disease prevalence

The commentary by Grunau and Linn (1) relating to an article of mine that was published in *Frontiers in Public Health—Epidemiology* (2) contains some important points, but I am left with a perception that the authors of that commentary have sometimes written with different frames of reference from the frame of reference within which I wrote my article. In this response, I will attempt to deal briefly with the areas where our frames of reference do and do not intersect and how our contributions might be used to maximum advantage.

Because the word limit for articles categorized as “perspectives” within *Frontiers in Public Health* (*FPH*) prevented me from writing a more detailed article originally, but also because of the stimulus provided by Grunau and Linn's commentary, in a separate publication (3) I have recently expanded on topics raised in my original article as well as on topics raised within Grunau and Linn's commentary.

Within this response, I deal briefly with the major topics that I think need to be addressed in direct response to Grunau and Linn's contribution, along with occasional reference to my subsequent article (3).

First, I believe it is important to acknowledge that the words *screening* and *diagnosis* carry different meanings for different people, as well, obviously, as inconsistent meanings for the same people. Therefore, in order to avoid confusion, I believe it is useful to clarify what is meant by these words in any particular context. I attempted to do that within the opening paragraph of the introduction in my original article, where I wrote:

Diagnostic tests are regarded as providing definitive information about the presence or absence of a target disease or condition. In contrast, screening tests—which are the focus of this article—typically have advantages over diagnostic tests such as placing fewer demands on the healthcare system and being more accessible as well as less invasive, less dangerous, less expensive, less time-consuming, and less physically and psychologically discomforting for clients. Screening tests are also, however, well-known for being imperfect and they are sometimes ambiguous. It is therefore important to determine the extent to which these tests are able to identify the likely presence or absence of a condition of interest so that their findings encourage appropriate decision making.

In my view, clear and consistent definitions concerning screening and diagnosis were not provided within Grunau and Linn's commentary. Indeed, there is confusing definitional slippage there, particularly between and within the words screening, diagnosis, and detection. I have attempted to provide greater clarity concerning these words in my subsequent article. That article appears in an open-access journal, so access to it should not be difficult.

Second, as pointed out by Wilson and Jungner in their seminal document published more than 50 years ago (4), the word screening can apply across seven contexts, ranging from contexts within conventionally conceived epidemiology (with its focus on the determinants, prevalence, incidence, and control of diseases *within a population*) to contexts that relate more to clinical epidemiology (with its focus on the prevention, identification, diagnosis, prognosis, and treatment of disease, as well as a focus on the accuracy of tests, *for individual people*).

Although I was remiss in not having been sufficiently clear within my original article that I was referring solely to the latter kind of situations, I believe that Grunau and Linn's commentary is disturbingly unclear because it moves from one situation to another without that being made obvious for readers. I have also attempted to address this kind of problem in my subsequent article.

Grunau and Linn correctly point out that prevalence can be conceived of in different ways when the performance of screening tests is being assessed. One of these they describe as “artificial” in that prevalence of a condition outside the specific context in which a test is being assessed (i.e., prevalence in the “real world”) is not necessarily taken into account.

In that so-called artificial situation, it is simply sufficient that two distinct groups of people are studied, with one group known to have the condition of interest and the other group known not to have that condition. This is the “standard” set of circumstances under which sensitivity and specificity are determined. The numbers of people in each group need not reflect the prevalence of the condition in the general population, or, even more pertinently, need not reflect the prevalence in a particular relevant subpopulation.

Grunau and Linn correctly point out that under these circumstances the calculations *related to predictive values* are almost inevitably inaccurate because population (or subpopulation) prevalence is not taken into account.

Although I had made reference to prevalence influencing predictive values in my original article, I acknowledge that I did not emphasize the issue sufficiently—although I attempted to alert readers to it on the second page of my article. There, in the paragraph that followed a set of calculations for determining sensitivity, specificity, and predictive values, I wrote:

The values that are entered into the cells of Figure 1 [i.e., the conventional diagram in which sensitivity, specificity, and predictive values are calculated] depend on … the prevalence of the target condition in the sample of people used in the analysis.

I acknowledge that this reference to the important issue of prevalence was very fleeting, but I believe that to have dealt with it in greater detail at that point in my manuscript would have been inappropriately distracting given other points I was making and for which I believed I needed to maintain a momentum.

I also referred to issues of prevalence in the following paragraph, where I wrote:

It might also be necessary to … ensure that there is a match between the samples that were used for assessing a screening test and the people subsequently being screened.

Because of the importance of that point, I provided five references in support of it, and readers could have gone to those articles had they sought further information. For the most part, the material in those references is clear and informative—as far as it goes.

Issues concerning prevalence are complex, however, and therefore I felt I could not do them justice given the word limit placed by *FPH* for submissions categorized as perspectives. In my subsequent article, I have provided what I regard to be appropriate information. There, on pages 68 to 70 (in no fewer than nine paragraphs containing more than 1,200 words of text), I have provided elaborated evidence indicating the need to take prevalence into account, but I have also extended my treatment of this topic by identifying difficulties in doing so.

One of the difficulties I emphasized in my follow-up article is that prevalence is not as easy to determine as might be anticipated, so the application of Bayes' theorem to obtain predictive values, as recommended by Grunau and Linn, might not provide a satisfactory basis for determining predictive values. Despite that, in my subsequent article, I demonstrated how Bayes' theorem is incorporated for obtaining predictive values for those who would appreciate that information.

In their commentary, Grunau and Linn argued that global, or summary, metrics such as the Youden index and the predictive summary index (PSI) are valid and useful global indicators of a screening test's credentials. As I have pointed out in my subsequent article, there is evidence that both of these metrics have limitations. For example, Šimundić (5) has argued that Youden's index is insensitive to differences in sensitivity and specificity, and Irving and Holden (6) have argued that the PSI is most effective when sensitivity and specificity are both high and prevalence is 50%, thus limiting its applicability because screening tests are typically unsatisfactory with regard to either sensitivity or specificity, or to both of those metrics, and, furthermore, prevalence of a condition is seldom 50%.

In addition, there seem to be no guidelines concerning when the Youden index and PSI can be regarded as indicating that a test is satisfactory, thus further depriving those metrics of usefulness.

In my subsequent article, I conducted a series of systematically sequenced analyses that demonstrate such global indices are not only limited but could be misleading.

This relates to a theme that I attempted to provide throughout my follow-up article, namely that it might be advisable if researchers and clinicians exhibit a degree of skepticism concerning metrics associated with screening tests. One outcome of this is my recommendation, by no means new, that the term *reference standard* be used instead of the term *gold standard*—a term that Grunau and Linn use in their commentary and that I believe contributes to conferring misplaced faith in metrics.

I am grateful for the commentary by Grunau and Linn because it gave me the fillip to write my follow-up article, and I hope that this response has provided rejoinders to their commentary that are helpful not only for clinicians and researchers, but also for others (including students) who are interested in understanding the salient issues—and there *do* seem to be people who are interested in these issues given that, within 2 years of its publication, my original article was viewed more than 32,750 times (more views than 99% of other *Frontiers* articles), and a little over 1 month later, at the time of writing, it has had more than 37,000 views.

## Author Contributions

The author confirms being the sole contributor of this work and has approved it for publication.

### Conflict of Interest

The author declares that the research was conducted in the absence of any commercial or financial relationships that could be construed as a potential conflict of interest.
